# In Vivo Fluorescence Molecular Imaging Using Covalent Organic Nanosheets Without Labeling

**DOI:** 10.1002/advs.202300462

**Published:** 2023-04-17

**Authors:** Seokmin Kang, Heesu Ahn, Chanho Park, Won Hyeok Yun, Ju Gyeong Jeong, Yong Jin Lee, Dong Wook Kim

**Affiliations:** ^1^ Department of Chemistry and Chemical Engineering Inha University 100 Inha‐ro Nam‐gu Incheon 22212 Republic of Korea; ^2^ Division of Applied RI Korea Institute of Radiological and Medical Sciences 75 Nowon‐ro Nowon‐gu Seoul 139706 Republic of Korea

**Keywords:** covalent organic frameworks, covalent organic nanosheets, fluorescence, in vivo imaging, in vivo imaging probes

## Abstract

Organic nanomaterials, as nanocarrier platforms, have tremendous potential for biomedical applications. The authors successfully prepared novel two‐dimensional covalent organic nanosheets (CONs) that can be used as efficient in vivo bioimaging probes by condensing 1,3,5‐triformylglucinol (Tp) and 2,7‐diaminopyrene (Py) to produce TpPy covalent organic frameworks (COFs). TpPy COFs are then subjected to a liquid exfoliation process to obtain TpPy CONs (< 200 nm in size and < 1.7 nm in thickness). TpPy CONs disperse well in water to provide a stable, homogeneous colloidal suspension, which shows favorable photoluminescence properties. Cell viability tests using MDA‐MB‐231 and RAW 264.7 cells reveal that TpPy CONs are low in cytotoxicity. Confocal microscopy reveals clear fluorescent cell images after incubation with TpPy CONs for 24 h, without reduction in cell activity or cytosolic aggregation. To investigate the biological behavior of the TpPy CONs, the authors perform an in vivo fluorescence imaging study using MDA‐MB‐231 tumor‐bearing mice. After intravenous injection of TpPy CONs disperse in phosphate‐buffered saline (PBS), persistent and strong fluorescence signals are observed in the tumor region, with low background signals from normal tissues at 1, 3, 12, and 24 h after injection. Furthermore, these in vivo imaging results concurred with ex vivo biodistribution and histological results.

## Graphical abstract

Thin‐layered covalent organic nanosheets (TpPy CONs) are easily produced by condensation of triformylglucinol (Tp) with diaminopyrene (Py) and followed by subsequent exfoliation. These nanosheets are well dispersed in water and phosphate‐buffered saline, showing favorable fluorescent properties and biocompatibility. They exhibit excellent performance as in vivo fluorescent molecular imaging probes in vivo and ex vivo.

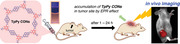

## 1 Introduction

Well‐designed nanoscale materials of optimal size have attracted considerable interest in nanomedicine as potential in vivo tumor bioimaging and therapeutic agents. This is because they can accumulate in tumor tissues owing to their enhanced permeability and retention (EPR) effects helping them to penetrate in tumor tissues.^[^ ^1^, ^2^, ^3^ ^]^ Noninvasive optical molecular imaging is a versatile and readily accessible imaging modality that can monitor biological processes in living subjects without any radiation penetration.^[^ ^4^, ^5^, ^6^ ^]^ In vivo optical imaging offers exciting opportunities for various biomedical applications, such as early diagnosis, treatment, and image‐guided surgery, owing to its suitability for real‐time analysis, rapid response, high sensitivity and specificity, and cost‐effectiveness.^[^ ^7^, ^8^, ^9^ ^]^ However, the successful application of this technique depends on the development of imaging probes (usually labeled with a low molecular‐weight organic dye) with suitable optical properties, high in vivo stability, biocompatibility, and low cytotoxicity.^[^ ^7^, ^10^, ^11^ ^]^

Semiconducting heavy metal‐based quantum dots (QDs) are considered as potential optical imaging probes because of their excellent photoluminescence (PL) without labeling and nanoscale dimensions.^[^ ^12^ ^]^ However, QDs have several intrinsic limitations, such as high cytotoxicity and poor dispersibility in aqueous media, which severely limit their use for molecular imaging studies.^[^ ^12^ ^]^

Thus, metal‐free nanocomposites‐based novel bioimaging probe frameworks with suitable optical and biological properties offer an attractive alternative. Several carbon‐based metal‐free nanocomposites, including carbon nanotubes, graphene derivatives, and carbon nitrides, have been investigated.^[^ ^3^, ^13^, ^14^, ^15^, ^16^, ^17^, ^18^ ^]^ Unfortunately, their poor optical properties and water dispersibility made them unlikely candidates for in vivo bioimaging probes.^[^ ^16^, ^18^ ^]^

Recently, covalent organic frameworks (COFs), a type of crystalline porous polymeric platform composed solely of organic building blocks, have received much interest for applications in optoelectronics, catalysis, energy storage, separation, and drug delivery because of their well‐defined structures and easily tailored properties.^[^ ^19^, ^20^, ^21^, ^22^, ^23^, ^24^, ^25^, ^26^ ^]^ Considering their inherent advantages, such as biocompatibility, tunable functionality, and unique stability, COFs have tremendous potential for biomedical applications.^[^ ^27^, ^28^ ^]^ Furthermore, 2D covalent organic nanosheets (CONs) can be obtained by exfoliation of COFs, such as self‐exfoliation, mechanical delamination, and solvent‐assisted exfoliation.^[^ ^16^, ^29^, ^30^, ^31^, ^32^, ^33^ ^]^ Exfoliation of COFs into CONs reduces their size as well as their *π*–*π* stacking interactions, thereby causing CONs to get separated into individual sheets to gain better dispersibility within the cells.^[^ ^34^, ^35^, ^36^ ^]^ However, several challenges still need to be overcome for in vivo bioimaging applications, as the optical properties of COFs and CONs developed to date are insufficient to overcome live tissue autofluorescence without labeling. Furthermore, the bioavailability of COFs is unsatisfactory in vivo owing to their poor water dispersibility and physiological stability.^[^ ^28^, ^33^, ^37^ ^]^

In this study, we developed a straightforward method for preparing a CON‐based nanoplatform (denoted as TpPy CONs) via the solvothermal condensation reaction and subsequent liquid exfoliation.^[^ ^38^, ^39^ ^]^ TpPy CONs showed excellent dispersibility, PL, and strong fluorescence in aqueous media.^[^ ^40^ ^]^ Furthermore, TpPy CONs produced highly stable and biocompatible suspensions, provided sufficient real‐time fluorescence responses to enable tracking in vivo, and efficiently accumulated in tumor tissues via the EPR effect after intravenous administration, which allowed tumor visualization without labeling or surface modification.

## 2 Results and Discussion

### 2.1 Preparation and Characterization of TpPy COF and CONs

**Figure 1:**
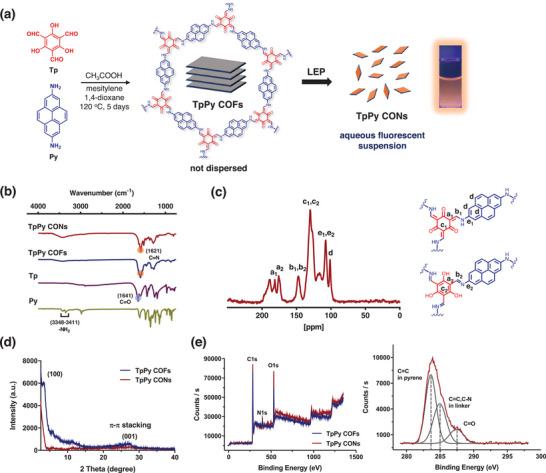
Synthetic routes and chemical characterizations for TpPy COFs and TpPy CONs. a) Schematic diagram of the preparation of TpPy CONs by solvothermal condensation and liquid exfoliation process (a fluorescent photograph of a 0.5 mg mL^−1^ TpPy CONs dispersion taken under 365 nm UV light). b) Stacked FT‐IR spectra of TpPy COFs, TpPy CONs, and their precursors; 2,4,6‐triformylphloroglucinol (Tp) and 2,7‐diaminopyrene (Py). c) ^13^C SSNMR spectrum of TpPy CONs (left) and chemical structures of tautomeric regions (right). d) XRD patterns of TpPy COFs and TpPy CONs. e) XPS survey spectra of TpPy COFs and CONs (left) and XPS C1s spectra including deconvolution of TpPy CONs (right).

The bulk material, referred to as TpPy COFs, was synthesized by Schiff condensation between 1,3,5‐triformylglucinol (Tp) and 2,7‐diaminopyrene (Py) under solvothermal conditions.^[^ ^26^, ^38^ ^]^ TpPy COF was isolated as a brownish powder, which was insoluble in water and all organic solvents, indicating the formation of stable polymeric materials. To achieve good water dispersibility for biomedical applications, liquid exfoliation processing (LEP) of TpPy COFs was performed to obtain the corresponding nanosheets (TpPy CONs) by ultrasonicating TpPy COFs in water for 5 h. The abundant hydrophilic groups (—NH_2_, —CHO, and —OH) were introduced by particle size reduction, and the edge surface area increased water dispersibility. TpPy‐CON produced stable and homogeneous colloidal suspensions that fluoresced strongly in water (**Figure** **1**a).

The formation of imine (C=N) linkages in the TpPy COFs and —CONs was confirmed by Fourier‐transform infrared (FT‐IR) spectroscopy in the presence of a strong absorption assigned to the C=N stretch at 1621 cm^−1^. The aldehyde (H—C=O) stretch absorption at 1641 cm^−1^ in Tp and the primary amine (—NH*_x_*) stretch absorption at 3348–3411 cm^−1^ in Py decreased significantly in TpPy COFs and —CONs because of the formation of imine linkages (Figure 1b).^[^ ^35^, ^38^, ^39^ ^]^ Broad peaks corresponding to —NH*_x_* stretching modes of the exfoliated CONs were more intense than those of the bulk COFs. FT‐IR data suggest that the TpPy CONs contained more edge structures compared to the bulk TpPy COFs. In addition, TpPy CONs had a larger absolute zeta potential (−35.1 mV) than that of the COFs (−10.7 mV), which was attributed to the increased numbers of “naked” *π*‐electrons and enol tautomer groups in its Tp moiety (Figure S1, Supporting Information).^[^ ^13^ ^]^ The chemical structures of these nanomaterials were also analyzed using solid‐state nuclear magnetic resonance (SSNMR) spectroscopy (Figure 1c). The ^13^C SSNMR spectrum of TpPy CONs revealed the presence of C atoms inside the C_d_ of the adjacent pyrene network (C_b1,2_ and C_e1,2_) bridging N atoms. Split peaks at C_a1,2_, C_b1,2_, C_c1,2_, and C_e1,2_ supported the presence of tautomerization in the TpPy CONs (Figure 1c, right), suggesting increased hydrophilicity and water‐dispersibility.^[^ ^30^ ^]^

Powder X‐ray diffraction (PXRD) analysis demonstrated that the as‐synthesized COFs and CONs materials were isostructural, with good crystallinity. The most intense peak at 2*θ* = 5° was assigned to the (100) plane, and the broad peak at 2*θ* = 27.5° corresponded to the reflection from the *π*–*π* stacked (001) plane (Figure 1d).^[^ ^19^ ^]^ The broad wide‐angle diffraction peaks were attributed to the sheet‐like layered morphology with an incomplete long‐range order of the TpPy COFs and CONs.^[^ ^35^ ^]^ The C1*s*, N1*s*, and O1*s* spectra of the TpPy CONs obtained from X‐ray photoelectron spectroscopy (XPS) were similar to those of the TpPy COFs (Figure 1e, left). The PXRD and XPS findings showed that the crystalline structure of the TpPy CONs was retained after exfoliation of the corresponding COFs. The C1*s* spectra of TpPy CONs showed a large peak at 283 eV, which was assigned to the C=C moieties of pyrene units, and the deconvoluted peaks at 285 and 288 eV, corresponded to C=N—C linkages and C=O groups in glucinol (Figure 1e; right). Thermogravimetric analysis (TGA) demonstrated excellent thermal stability of TpPy COFs and CONs, which exhibited a weight loss of < 40% at 800°C (Figure S2, Supporting Information).

**Figure 2:**
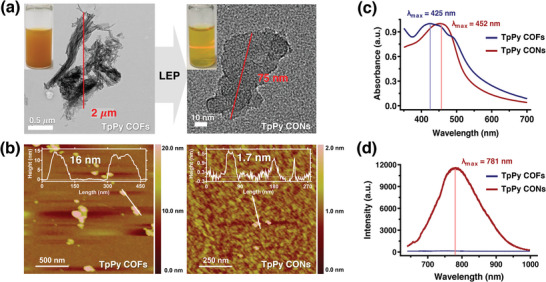
a) TEM images of TpPy COFs (left) and TpPy CONs (right). Insets show photographs of aqueous colloidal suspensions of TpPy COFs and CONs showing the Tyndall effect with concentration of 0.5 mg mL^−1^. b) AFM images of TpPy COFs (left) and CONs (right). c) UV–vis adsorption and d) PL spectra of TpPy COFs and CONs suspensions. Photoexcitation was performed using a 633 nm He–Ne laser.

The optimum particle size for efficient tumor cellular uptake is < 200 nm,^[^ ^28^ ^]^ and thus, the changes in morphological properties from TpPy COFs to CONs were investigated by transmission electron microscopy (TEM) and atomic force microscopy (AFM). As depicted in **Figure** **2**a, TEM images showed that the sheet size was reduced by exfoliation from 1–2 µm to < 200 nm, which supported hydrodynamic sizes determined by dynamic light scattering (DLS) measurements (Figure S3, Supporting Information) and average particle sizes determined by TEM. AFM revealed that TpPy CONs had a 2D sheet‐like morphology. The thickness of the 2D nanosheets was <1.7 nm, indicating that layer separation occurred during exfoliation, as the TpPy COFs had a thickness of ≈16 nm. TpPy CONs suspension, unlike TpPy COFs suspension, showed a distinct Tyndall effect, which confirmed the presence of highly monodisperse nanosheets (inset in Figure 2a).

Next, we investigated the changes in the optical properties caused by exfoliation using UV–vis absorption and PL spectroscopy. As depicted in Figure 2c, broadband adsorptions were observed in the wide visible light region, indicating laminated structures with enhanced light harvesting capacity in the visible light region due to the widely delocalized *π* electrons of these materials.^[^ ^41^ ^]^ Aqueous dispersions of TpPy CONs exhibited a maximum absorption (*λ*_max_) at 452 nm, which was redshifted compared to that of TpPy COFs (*λ*_max_ = 425 nm). In addition, this adsorption peak was narrower for the TpPy CONs, indicating that the light‐adsorbing unit homogeneity was marginally improved by exfoliation.^[^ ^13^ ^]^ In the PL spectra (Figure 2d), when excitation occurred at 633 nm, the TpPy CONs dispersions exhibited strong emission at 781 nm, whereas the TpPy COFs dispersions did not. The poor emissive properties of TpPy COFs may have been due to aggregation‐induced quenching,^[^ ^42^ ^]^ indicating that TpPy CONs exhibited markedly better emission owing to reduced interlayer *π*–*π* interactions. Density functional theory (DFT) calculations showed that the excellent PL properties of TpPy CONs may also be due to intramolecular donor‐acceptor charge transfer from pyrene groups (donors) to the central glucinol moieties (acceptors) (Figure S5, Supporting Information).^[^ ^43^ ^]^

### 2.2 In Vitro Study of TpPy CONs

**Figure 3:**
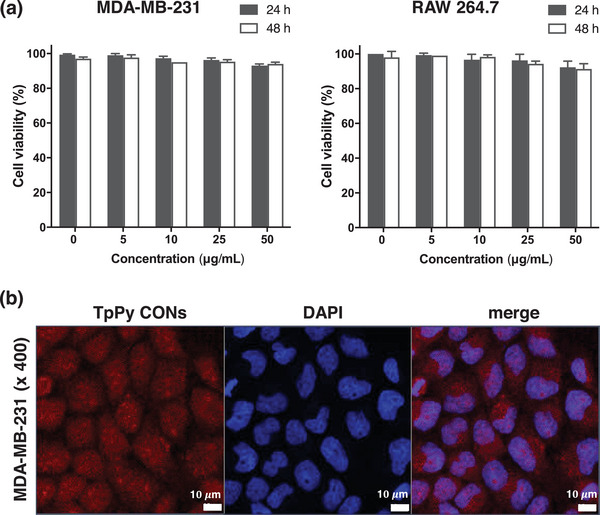
In vitro study of TpPy CONs. a) Cell viability of MDA‐MB‐231 and RAW 264.7 cells after incubation for 24 or 48 h with different concentrations of TpPy CONs as determined by a trypan blue exclusion assay. Results are presented as means ± SDs (*n* = 3 for each group). b) A confocal fluorescence microscopic image of MDA‐MB‐231 cells after incubation for 24 h at 37 °C with TpPy CONs at 5 µg mL^−1^ (left), cell nucleus images after DAPI staining (center), and a merged image (right). Original magnification 400x. Scale bar = 10 µm.

To use TpPy CONs as an optical/fluorescence bioimaging probe in vitro and in vivo, their biocompatibility and cytotoxicity was evaluated by incubating MDA‐MB‐231 and RAW 264.7 cells in 0, 5, 10, 25, or 50 µg mL^−1^ dispersions of TpPy CONs for 24 and 48 h using a trypan blue exclusion assay. At 5–25 µg mL^−1^, TpPy CONs barely inhibited the proliferation of MDA‐MB‐231 or RAW 264.7, after incubation for 24 or 48 h (**Figure** **3**a). In addition, after incubation for 48 h at the highest concentration examined (50 µg mL^−1^), TpPy CONs only reduced the viability of MDA‐MB‐231 and RAW 264.7 cells to 94.0 ± 1.0 and 91.3 ± 3.1%, respectively.

In vitro cell imaging by confocal fluorescence microscopy was performed using MDA‐MB‐231 cells with well‐dispersible TpPy CONs in biological media to investigate the possibility of cell labeling using TpPy CONs (Figure 3b). MDA‐MB‐231 cells treated with TpPy CONs for 24 h maintained their morphology and activity, providing clear cell images (left image in Figure 3b) with no evidence of cytosolic aggregation. The images show that the red‐emitting TpPy nanosheets were internalized into MDA‐MB‐231 cells. Notably, TpPy CONs were well‐visualized in cells treated with only 5 µg mL^−1^, owing to their favorable PL properties. In addition, merged DAPI‐stained cell images (right image in Figure 3b) confirmed that the cells were labeled successfully by TpPy CONs. These encouraging in vitro cell viability and confocal imaging results led to perform an in vivo study.

### 2.3 In Vivo Molecular Imaging Study Using TpPy CONs in Tumor Models

**Figure 4:**
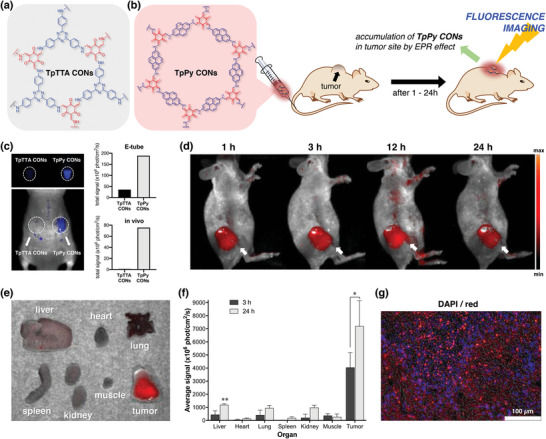
In vivo fluorescence imaging and *ex vivo* biodistribution. a) Chemical structure of TpTTA CONs used as using a control group. b) Schematic illustration of the procedure for in vivo fluorescence imaging using TpPy CONs. c) Comparison of the fluorescent intensity of TpTTA CONs and TpPy CONs as determined by the e‐tube test (upper image), and in vivo imaging (lower images) of a nude mouse subcutaneously injected with 86 µg of TpTTA CONs (left side) and TpPy CONs (right side). d) Representative in vivo fluorescence images of MDA‐MB‐231 tumor‐bearing mice intravenously injected via a tail vein with 15 µg of TpPy CONs recorded at 1, 3, 12, and 24 h after injection. White arrows indicate the tumor position. e) Representative ex vivo fluorescence image of the major organs from an MDA‐MB‐231 tumor‐bearing mouse at 24 h after TpPy CONs injection. f) Quantified necropsy data. Mean TpPy CONs fluorescence intensity of necropsied major organs and tumor, measured at 3 and 24 h after injection. Results are presented as means ± SDs (*n* = 4 for each group). * *p* < 0.05 (at 3 vs 24 h in tumor) and ** *p* < 0.01 (liver vs tumor at 24 h). g) Representative histological fluorescent microscopic image of tumor tissue sections from an MDA‐MB‐231 tumor‐bearing mouse sacrificed at 24 h after TpPy CONs administration. Original magnification 100x. Scale bar = 100 µm.

For in vivo fluorescent molecular imaging study, the fluorescent intensities of TpPy CONs were evaluated in vitro and in vivo using a fluorescence imaging system (**Figure** **4**c) and compared with those of TpTTA CONs (Figure 4a) as a control, which were prepared from Tp and TTA (4,4′,4″‐(1,3,5‐triazine‐2,4,6‐triyl)trianiline) instead of Py. In the e‐tube test (Figure 4c, top), TpPy‐CON had an ≈5‐fold higher fluorescent signal intensity than obtained with TpTTA CONs. To determine whether live tissue autofluorescence interfered with the fluorescence signals, an in vivo fluorescence imaging study was conducted using female Balb/c mice subcutaneously injected with TpPy CONs (right side) and TpTTA CONs (left side). As shown in Figure 4c (bottom), TpPy CONs provided a 70‐fold stronger signal than that obtained with TpTTA CONs.

Owing to the excellent results obtained for TpPy CONs with respect to optimal size, enhanced PL characteristics, dispersibility in biological media, low cytotoxicity, and biocompatibility, an in vivo bioimaging study using them as an optical/fluorescent probe was performed. TpPy CONs (15 µg) were dispersed in phosphate‐buffered saline (PBS) and intravenously injected via the tail vein into mice with a xenograft MDA‐MB‐231 tumor on the right thigh (Figure 4d). In vivo fluorescence images obtained at 1, 3, 12, and 24 h after injection showed strong fluorescence signals in tumor sites from 1 h after injection without any interference from live tissue autofluorescence, which indicated that TpPy CONs rapidly accumulated in tumor tissues by passive targeting and the EPR effect. Moreover, persistent high fluorescent signals were observed in tumors with low background signals from normal tissues at all intervals after administering the 2D TpPy CONs. These in vivo fluorescence imaging results demonstrate that TpPy CONs can be used to visualize tumors rapidly without any labeling procedure.

### 2.4 Ex Vivo Study Using TpPy CONs

To confirm in vivo fluorescence imaging results, the biodistribution of TpPy CONs was tested by directly tracking their intrinsic fluorescence in organs ex vivo from necropsied mice at 24 h after injection. As indicated by the ex vivo images in Figure 4e, a much stronger fluorescence intensity of TpPy CONs was detected in tumor tissue than in other major organs, such as the liver, heart, lung, spleen, kidney, and muscle, showing a good correlation with the in vivo fluorescence imaging results. Furthermore, quantitative analysis showed well‐matched ex vivo fluorescence intensities in the major organs and tumor tissues (Figure 4f). Notably, ex vivo fluorescence intensities of tumor tissues were 19–167‐fold higher than those of the heart, spleen, kidney, and muscle, which adequately demonstrated the extremely high tumor uptake of TpPy CONs achieved by passive tumor targeting and the EPR effect. Further ex vivo quantitative analysis at 3 and 24 h after injection (Figure S7, Supporting Information) revealed that the tumor‐to‐liver and tumor‐to‐lung ratio of fluorescence intensity decreased over time (from 3 to 24 h after injection), whereas the tumor‐to‐muscle ratio increased significantly, suggesting that the clearance of TpPy CONs was related to the reticuloendothelial system (RES).^[^ ^44^ ^]^

### 2.5 Histological Analysis

Next, we performed a histological study using a fluorescence microscope to confirm the accretion of TpPy CONs into the tumor tissue. As shown in this microscopic image (Figure 4g), bright fluorescence spots were observed clearly in tumor sections of the sacrificed tumor‐bearing mice 24 h after TpPy CONs administration, which indicates that these administered nanosheets were well taken up by the tumor cells. Therefore, the ex vivo biodistribution and histological results concurred with the in vivo fluorescence imaging results.

## 3 Conclusion

In summary, we have successfully synthesized novel thin‐layered covalent organic molecule‐based nanosheets as metal‐free, biocompatible, fluorescent bioimaging probes. To prepare TpPy CONs, bulk TpPy COFs were initially synthesized by the condensation reaction of Tp with Py, and subsequently, when subjected to a liquid exfoliation process, they produced a stable and homogeneous colloidal 2D TpPy CONs suspension (< 200 nm in size with a 1.7 nm thickness). TpPy CONs dispersed easily in biological media, exhibited low cytotoxicity, high biocompatibility, and excellent PL properties, and produced strong fluorescence in aqueous media. In vitro confocal imaging demonstrated that the fluorescent TpPy CONs suspension provided clear cell images without any evidence of cytosolic aggregation or weakening of cell activity. Furthermore, in vivo optical imaging of tumor‐bearing mice administered with TpPy CONs showed persistent strong fluorescence signals from tumor lesions without any interference from live tissue‐induced autofluorescence. In addition, the animal study revealed that TpPy CONs visualized tumors well and produced sufficient real‐time fluorescence response to enable tracking in vivo without the need for fluorescent tagging. We hope that “label‐free” TpPy CONs will be useful in many biomedical applications. Further studies on the theranostic applications of 2D nanosheet tracking are underway.

## 4 Experimental Section

### Preparation of TpPy COFs

In a vial (5 mL), 0.2 mmol of 1,3,5‐triformylphloroglucinol (Tp), 0.3 mmol of 2,7‐diaminopyrene (Py), 1 mL of 1,4‐dioxane, 1 mL of mesitylene, and 0.16 mL of 0.6 m acetic acid were added. The mixture was sonicated for 1 min until homogeneous and then capped, flash frozen at 77 K (in a liquid nitrogen bath), degassed by freeze‐pump‐thawing several times, and heated at 120 °C in an oil bath for 5 days. The resulting precipitate (TpPy COFs) was collected as a brownish powder by centrifugation and washed sequentially with acetone, chloroform, THF, and H_2_O.

### Production of Aqueous Suspensions of TpPy CONs

TpPy CONs were obtained by exfoliation of TpPy COFs. TpPy COF powder (3 mg 10 mL^−1^ of water) was added to a vial filled with deionized water and sonicated for 200 min using an ultrasonic cleaner (POWERsonic 410, Hwa‐Shin Instrument Co., South Korea). To remove heavy particles, the suspension was centrifuged for 2 min, and the supernatant (TpPy CONs suspension) was collected.

### In Vitro Cell Viability Test

TpPy CONs at 0, 5, 10, 25, or 50 µg mL^−1^ in PBS were added to 1 × 10^6^ MDA‐MB‐231 or RAW 264.7 cells in 1 mL of cell culture media and incubated at 37 °C for 1 h. After centrifugation at 1000 rpm for 5 min, the supernatants were removed, and the cells were washed thrice with PBS. TpPy CONs labeled MDA‐MB‐231 cells, or RAW 264.7 cells, were plated and incubated in Dulbecco's Modified Eagle Medium (DMEM) containing 10% fetal bovine serum (FBS) at 37 °C in a humidified 5% CO_2_ atmosphere for 24 or 48 h. The viability of TpPy CONs labeled cell lines was determined using the trypan blue exclusion test (*n* = 3). Results are presented as mean ± standard deviation (SDs).

### Confocal Fluorescent Microscopy Image Study

MDA‐MB‐231 cells (1 × 10^4^) were seeded in 2‐well chamber slides in 1 mL of DMEM media (containing 10% FBS and 1% antibiotics) per well and incubated at 37 °C in a humidified 5% CO_2_ atmosphere for 24 h. The cells were then treated with TpPy CONs dissolved in DMEM (5 µg mL^−1^) and incubated under the same conditions for 24 h. After removing the supernatant, cells were washed twice with PBS, treated with 0.5% Triton X‐100, washed with PBS, and stained with DAPI. Fluorescence images were obtained using a laser‐scanning confocal microscope (LSM 800; Carl Zeiss MicroImaging, Jena, Germany) at 400x.

### Fluorescence Imaging of TpTTA CONs and TpPy CONs

The fluorescence intensities of TpTTA CONs and TpPy CONs were compared using the Maestro In vivo Imaging System (Califer Life science Inc, MA). TpTTA and TpPy CONs (86 µg) were dissolved in PBS and placed in an e‐tube. Fluorescence imaging was performed using a Maestro In Vivo Imaging System (excitation = 455 nm, emission = 620 nm). Subsequently, each suspension of TpTTA CONs and TpPy CONs was subcutaneously injected into the dorsal skin of 6‐week‐old female Balb/c mice at a depth of 2 mm under 2% isoflurane in oxygen anesthesia. Fluorescence images were obtained immediately using a Maestro in vivo imaging system. The total fluorescence intensity was obtained by drawing regions of interest (ROIs).

### In Vivo and Ex Vivo Fluorescence Imaging in MDA‐MB‐231 Tumor‐Bearing Mice

All animal experimental procedures were approved by the Institutional Animal Care and Use Committee (IACUC) of the Korea Institute of Radiological and Medical Sciences (KIRAMS) (no. KIRAMS 2018‐0064). MDA‐MB‐231 tumor‐bearing mice (BALB/c nude, female, 6 weeks, *n* = 4) were intravenously administered with 15 µg of TpPy CONs under 2% isoflurane in oxygen anesthesia. In vivo fluorescence images were acquired at 1, 3, 12, and 24 h after injection under 2% isoflurane in oxygen anesthesia using the Maestro in vivo imaging system (excitation = 455 nm, emission = 620 nm). Subsequently, the mice were necropsied, and major organs (liver, heart, lung, spleen, kidneys, and muscle) and tumors were harvested for ex vivo imaging at 3 and 24 h after injection. Average fluorescent signals in the major organs were obtained by drawing ROIs.

### Histological Analysis

After acquiring ex vivo imaging, MDA‐MB‐231 tumor tissues were fixed in 4% PFA and frozen with optimal cutting temperature (OCT) compound (Leica, Wetzlar, Germany) at −20 °C. Tumor tissues were then serially sectioned at 5 µm using a cryostat (Cryocut 1950, Leica, Wetzlar, Germany), placed on silane‐coated glass slides (MUTO, Tokyo, Japan), washed with PBS, and stained with ProLong Diamond Antifade Mountant with DAPI (Invitrogen, Carlsbad, CA). Sections were then cover‐slipped (Paul Marienfeld, Harsewinkel, Germany), and fluorescence images were obtained using a confocal microscope (LSM 800, Carl Zeiss MicroImaging, Jena, Germany) at 100x.

### Statistical Analysis

In vivo and ex vivo experimental statistical data were analyzed from at least four samples and expressed as mean ± SD. Statistically significant differences were determined with the unpaired Student`s *t*‐test with GraphPad Prism software (ver. 5.0). *p* values were considered to be statistically significant (* *p* < 0.05 and ** *p* < 0.01).

## Conflict of Interest

The authors declare no conflict of interest.

## Supporting information

Supporting Information

## Data Availability

The data that support the findings of this study are available in the supplementary material of this article.
