# Supplementary material for: In Vivo Fluorescence Molecular Imaging Using Covalent Organic Nanosheets Without Labeling
Source: Adv Sci (Weinh). 2023 Apr 17;10(16):2300462. doi: 10.1002/advs.202300462 (PMC10238211; doi:10.1002/advs.202300462)
Supplement: Supplementary file 1 — Supporting Information [file ADVS-10-2300462-s001.pdf]

ADVANCED SCIENCE
Open Access

Supporting Information

for *Adv. Sci.*, DOI 10.1002/advs.202300462

In Vivo Fluorescence Molecular Imaging Using Covalent Organic Nanosheets Without Labeling

Seokmin Kang, Heesu Ahn, Chanho Park, Won Hyeok Yun, Ju Gyeong Jeong, Yong Jin Lee and Dong Wook Kim**

Supporting Information

In Vivo Fluorescence Molecular Imaging Using Covalent Organic Nanosheets without Labeling

Seokmin Kang,† Heesu Ahn,† Chanho Park, Won Hyeok Yun, Ju Gyeong Jeong, Yong Jin Lee, and Dong Wook Kim**

Preparation of TpPy COFs. A vial (5 mL) was charged with 0.2 mmol of 1,3,5-triformylphloroglucinol (Tp), 0.3 mmol of 2,7-diaminopyrene (Py), 1mL of 1,4-dioxane, 1mL of mesitylene, and 0.16 mL of 0.6 M acetic acid. The mixture was sonicated for 1 min until homogeneous and then capped, flash frozen at 77 K (in a liquid nitrogen bath), degassed by freeze-pump-thawing several times, and heated at 120 °C in an oil bath for 5 days. The resulting precipitate (TpPy COFs) was collected as a brownish powder by centrifugation and washed sequentially with acetone, chloroform, THF, and H_2O.

Production of aqueous suspensions of TpPy CONs. TpPy CONs were obtained by the exfoliation of TpPy COFs. The TpPy COF powder (3 mg per 10 mL of water) was added into a vial filled with de-ionized water and sonicated for 200 min using an ultrasonic cleaner (POWERsonic 410, Hwa-Shin Instrument Co., South Korea). To remove heavy particles, the suspension was centrifuged for 2 min, and the supernatant (TpPy CONs suspension) was collected.

Characterization. X-Ray photoelectron spectroscopy (XPS) was performed using an angle-resolved X-ray photoelectron MXR1 Gun spectrometer operated at 400 µm and 15 kV (Theta probe, Thermo Fisher Scientific, UK); the analysis was performed with sample attached by copper tape and binding energies were determined versus the N 1*s* peak at 398.8 eV. Elemental analysis was conducted using a FLASH EA1112 instrument (Thermo Electron, Italy) for C, N, and H. X-ray diffraction (XRD) patterns were obtained using a DMAX-2500 unit (Rigaku, Tokyo, Japan). Photoluminescence (PL) spectra were obtained using a spectrograph (f = 0.5 m,

Spectrograph 500i, Acton Research Co., USA) equipped with an intensified CCD (PI-MAX3, Princeton Instruments, IRY1024, USA) and He-Cd 325 nm laser. Fourier-transform infrared (FT-IR) spectra of samples in KBr pellets were obtained using 64 scans of an FT-IR vacuum spectrometer (VERTEX 80V, Bruker, Germany). Transmission electron microscopy (TEM) images were obtained using a JEOL JEM2100F instrument (JEOL Co. Ltd., Japan) at 200 kV. Individual samples were prepared for TEM analysis by drying a droplet of a mixture including TpPy COFs or TpPy CONs powder in water on a carbon-coated copper grid (LC300-Cu, Electron Microscopy Sciences). A field-emission gun (S-4300SE, Hitachi, Japan) provided the field-emission scanning electron microscope (SEM) images. ^{13}C solid state nuclear magnetic resonance (SSNMR) spectra were acquired from a solid state NMR (SSNMR) spectrometer (400 MHz, AVANCE III HD, Bruker, Germany) at KBSI Western Seoul Center with an H/X CPMAS probe equipped with a 4 mm zirconia rotor at room temperature at 100 MHz. UV-vis diffuse reflectance absorption spectra were obtained using a UV-2600 instrument (Shimadzu, Japan) equipped with an ISR-2600 Plus integrating sphere attachment. Atomic force microscope (AFM) images were provided by using a XE-100 microscope (PSIA, Korea). The zeta potential was measured in aqueous dispersion using a zeta potentiometer (ELS-Z, Otsuka Electronics).

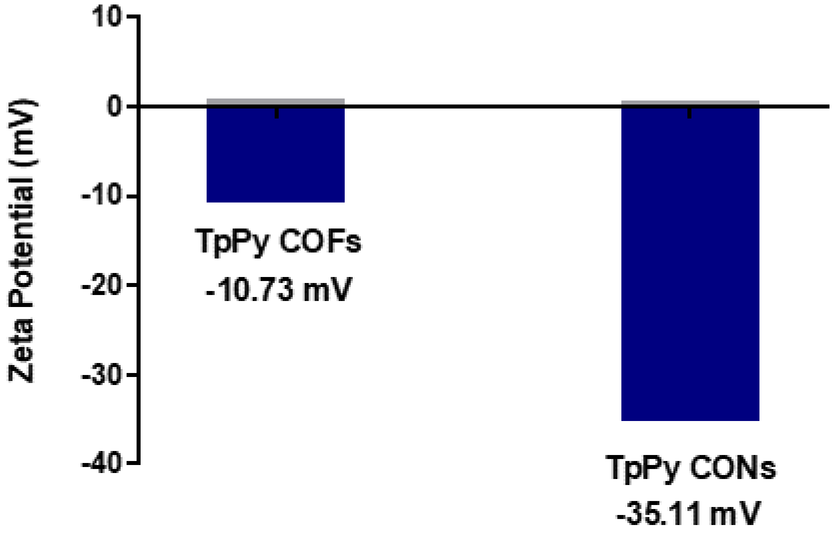

Figure S1. Zeta potentials of TpPy COFs and TpPy CONs.

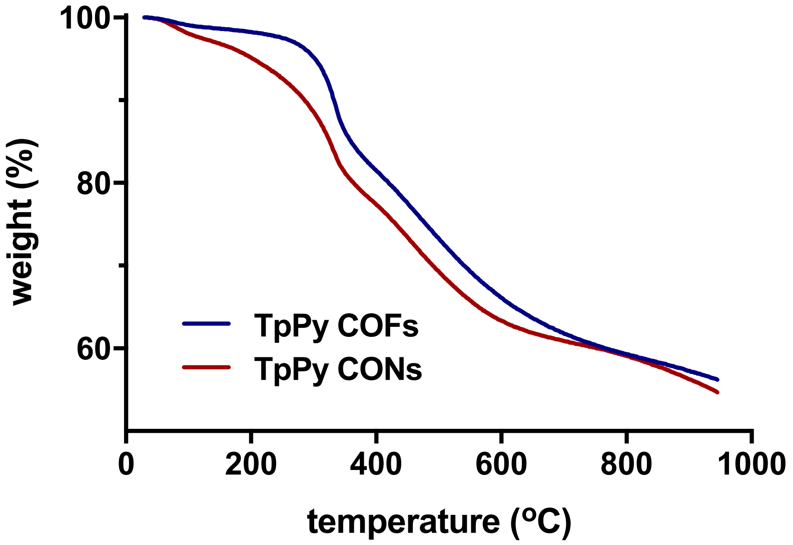

Figure S2. TGA curves of TpPy COFs and TpPy CONs under air atmosphere

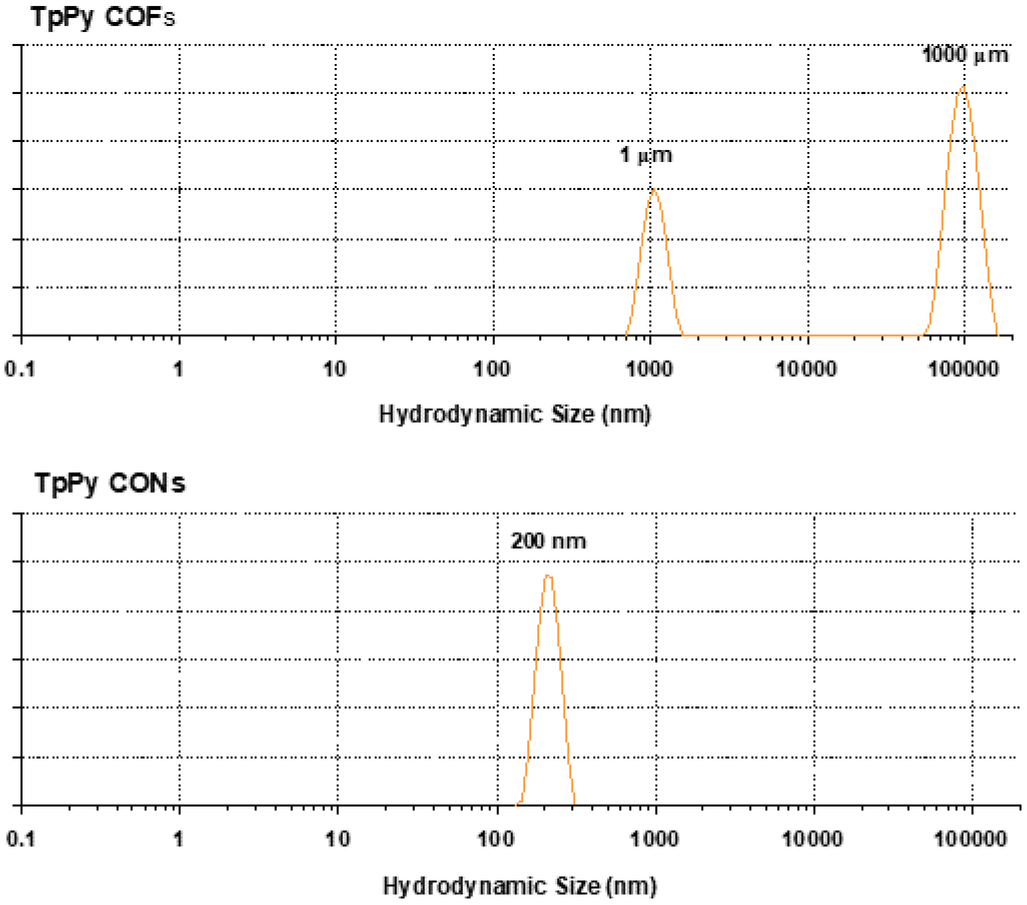

Figure S3. Hydrodynamic diameter of TpPy COFs (up) and TpPy CONs (down) in deionized water measured by dynamic light scattering (DLS).

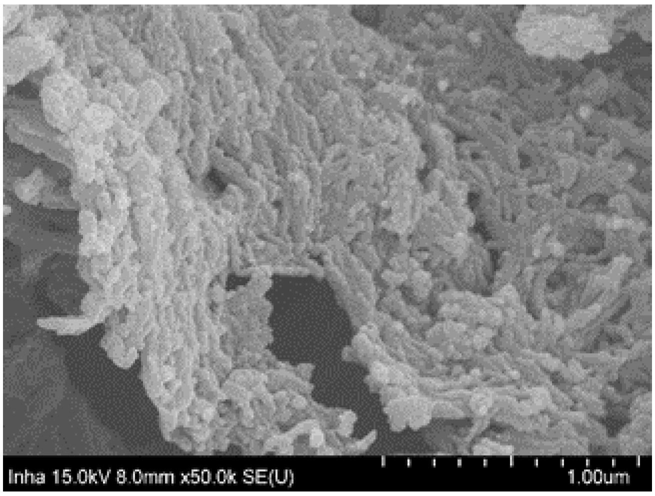

Figure S4. SEM image of TpPy CONs.

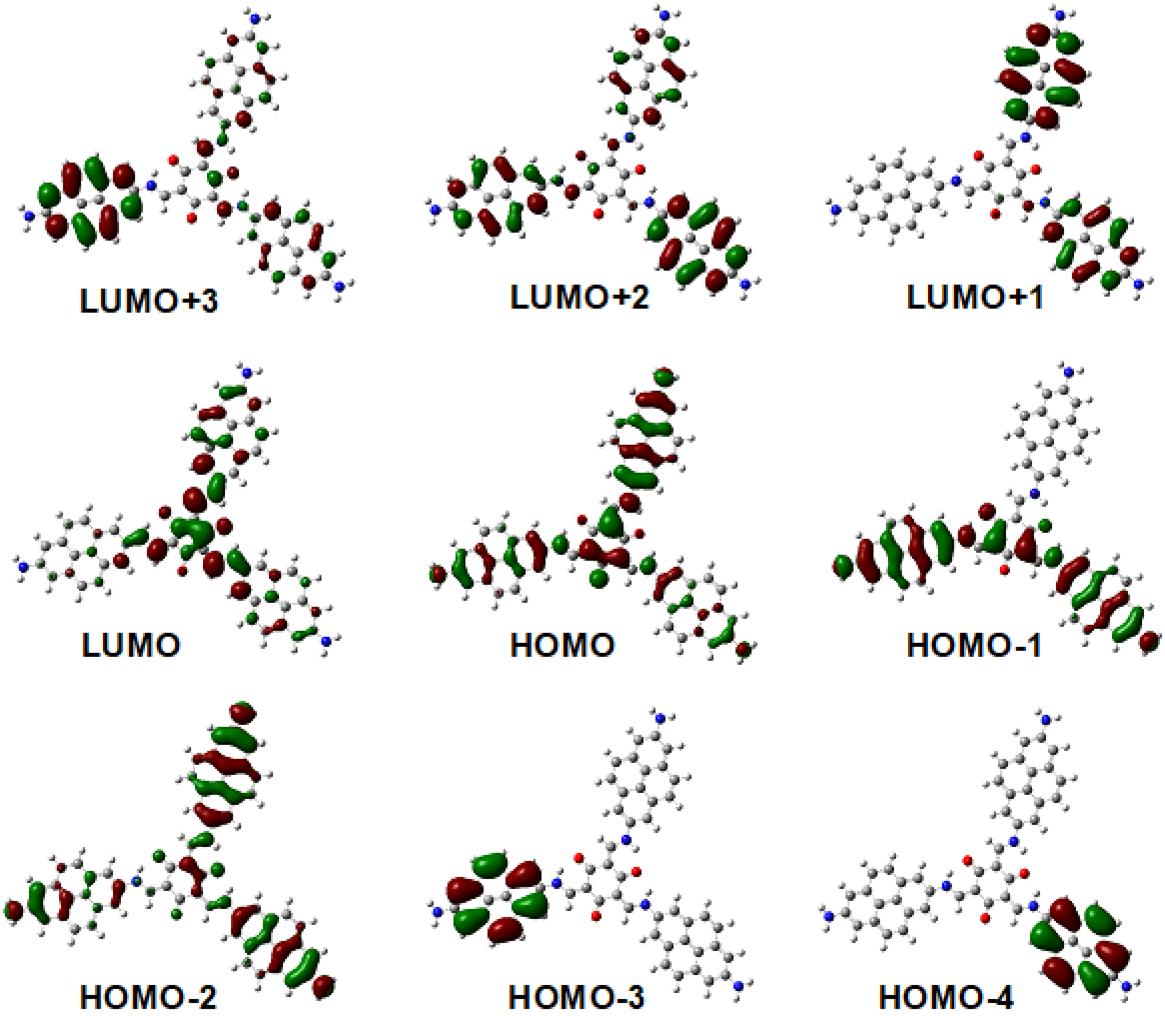

Figure S5. HOMO and LUMO energy diagram of partial structure of TpPy CONs calculated from density functional theory, DFT (3-21G) basis set.

***In vitro* cell viability test (Figure 3a).**

TpPy CONs at 0, 5, 10, 25, or 50 μg/mL in phosphate buffered saline (PBS) was added to 1× 10^6 MDA-MB-231 or RAW 264.7 cells in 1 mL of cell culture media and incubated at 37 °C for 1 hr. After centrifugation at 1000 rpm for 5 min, supernatants were removed, and the cells were washed three times with PBS. TpPy CONs labelled MDA-MB-231 cells or RAW 264.7 cells were plated and incubated in DMEM containing 10% FBS at 37 °C in a humidified 5% CO_2 atmosphere for 24 or 48 hr. The viabilities of TpPy CONs labeled cell lines were determined using the trypan blue exclusion test. Results are presented as means±SDs.

Confocal fluorescent microscopy image study (Figure 3b)

MDA-MB-231 cells (1×10^4) were seeded in 2-well chamber slides in 1 mL of DMEM media (containing 10% FBS and 1% antibiotics) per well and incubated at 37 °C in a humidified 5% CO_2 atmosphere for 24 hr. Cells were then treated with TpPy CONs dissolved in DMEM media (5 ug/mL) and incubated under the same conditions for 24 hr. After removing the supernatant, cells were washed twice with PBS, treated with 0.5 % Triton X-100, washed with PBS, and stained with DAPI. Fluorescence images were obtained using a laser-scanning confocal microscope (LSM 800, Carl Zeiss MicroImaging, Jena, Germany) at 400x.

Fluorescence imaging of TpTTA CONs and TpPy CONs (Figure 4c)

The fluorescence intensities of TpTTA CONs and TpPy CONs were compared using the Maestro *In vivo* Imaging System (Califer Life science Inc, Massachusetts, USA). TpTTA CONs and TpPy CONs (86 μg) were dissolved in PBS and contained in an e-tube. The fluorescence imaging was obtained by Maestro In Vivo Imaging System (excitation = 455 nm, emission = 620 nm). Subsequently, each suspension of TpTTA CONs and TpPy CONs was subcutaneously injected into dorsal skins of 6-week-old female, Balb/c mice at a depth of 2 mm, under 2% isoflurane in oxygen anesthesia. Fluorescence images were immediately obtained using the Maestro *In vivo* Imaging System. Total fluorescence intensities were obtained by drawing regions of interest (ROIs).

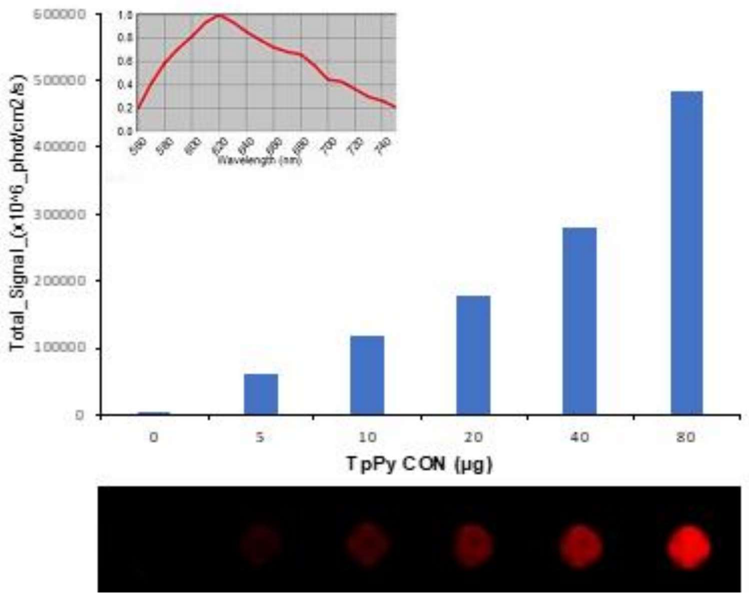

Figure S6. Fluorescence profile of TpPy CONs (inset) and their concentration dependent fluorescence intensities obtained from the Maestro *In vivo* Imaging System.

In vivo and _ex vivo_ fluorescence imaging in MDA-MB-231 tumor-bearing mice (Figure 4d-f)

All animal experiments procedures were approved beforehand by the Institutional Animal Care and Use Committee (IACUC) at the Korea Institute of Radiological and Medical Sciences (KIRAMS) (no. KIRAMS 2018-0064). MDA-MB-231 tumor-bearing mice (Balb/c, nude, female, 6 weeks, n = 4) were intravenously administrated with 15 µg of TpPy CONs under 2% isoflurane in oxygen anesthesia. *In vivo* fluorescence images were acquired at 1 h, 3 h, 12 h, and 24 h after injection under 2% isoflurane in oxygen anesthesia using the Maestro *In vivo* Imaging System (excitation = 455 nm, emission = 620 nm). Subsequently, mice were necropsied, and major organs (liver, heart, lung, spleen, kidneys, and muscle) and tumor were harvested for *ex vivo* imaging at 3 and 24 h after injection. Average fluorescent signals in major organs were obtained by drawing ROIs.

Histological analysis (Figure 4g)

After acquiring ex vivo imaging, MDA-MB-231 tumor tissues were fixed in 4 % PFA, and frozen with optimal cutting temperature (O.C.T) compound (Leica, Wetzlar, Germany) at –20 °C. Tumor tissues were then serially sectioned at 5 µm using a cryostat (Cryocut 1950, Leica, Wetzlar, Germany), placed on silane-coated slide glass (MUTO, Tokyo, Japan), washed with PBS, and stained with ProLong™ Diamond Antifade Mountant with DAPI (Invitrogen, California, USA). Sections were then coverslipped (Paul Marienfeld, Harsewinkel, Germany),

and fluorescence images were obtained using a confocal microscope (LSM 800, Carl Zeiss MicroImaging, Jena, Germany) at 100x.

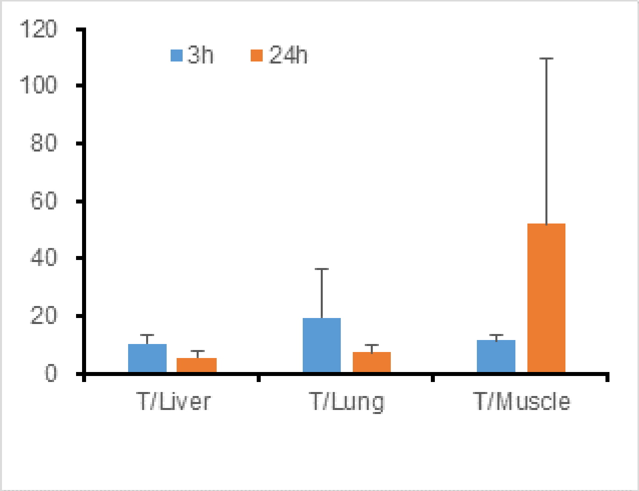

Figure S7. Tumor to liver, tumor to lung, and tumor to muscle ratio at 3 and 24 h after injection from *ex vivo* analysis.

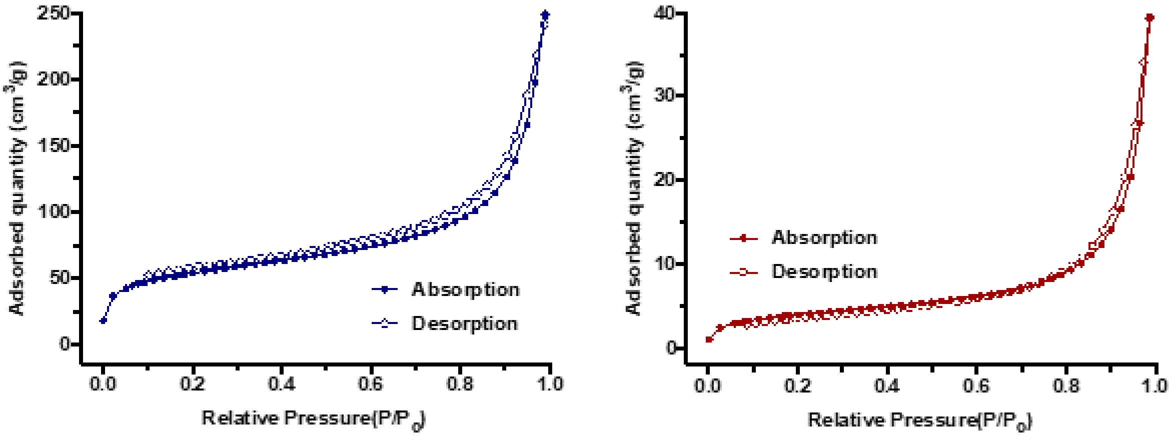

Figure S8. N_2 absorption and desorption isotherm at 77 K of TpPy COF (left) and CONs (right).
